# Pace and shape of senescence in three species of duckweed

**DOI:** 10.1002/ece3.9038

**Published:** 2022-07-05

**Authors:** Austin P. Paiha, Robert A. Laird

**Affiliations:** ^1^ Department of Biological Sciences University of Lethbridge Lethbridge Alberta Canada

**Keywords:** aging, aquatic plants, Araceae, demography, interspecific comparison, *Lemna*, Lemnaceae, Lemnoideae, survivorship

## Abstract

Senescence is progressive bodily deterioration associated with declines in survival and fecundity in older age classes. There is great diversity in patterns of senescence across species, but these patterns can be difficult to compare formally due to variation in the absolute time scales in which species live and die: members of some species live for a matter of days, others for millennia. To address this issue, the “pace‐shape” approach was developed to decouple absolute time from analyses and instead standardize life history traits in terms of average life expectancy, facilitating intra‐ and interspecific comparisons. Here, we use this approach to distinguish the generic form of demographic trajectories (shape) from the time scale on which the trajectories occurred (pace) in three species of tiny, free‐floating aquatic plants known as duckweeds (*Lemna gibba* L., *L. minor* L., and *L. turionifera* Landolt), which have mean lifespans of less than a month under typical lab conditions, and exhibit age‐related declines in survivorship and reproduction. Using a randomized block design in which we tracked a final total of 430 individuals, we report differences in pace and shape among the three species. Specifically, the largest, least‐fecund, and typically longest‐lived species, *L. gibba*, tended to exhibit more rapid decreases in time‐standardized survivorship and fecundity compared with the other two species. This study emphasizes variation in aging patterns that can be found among plant species, including those in the same genus, and provides further validation for the utility of applying the pace and shape approach in interspecific comparisons.

## Graphical abstract

Senescence is progressive bodily deterioration associated with declines in survival and fecundity in older age classes. We tracked three species of aquatic plants in the genus *Lemna* to compare their pace and shape of senescence. The largest, least‐fecund, and typically longest‐lived species, *Lemna gibba* (LG), tended to exhibit more rapid decreases in time‐standardized survivorship and fecundity compared to the other two species, *L. minor* (LM) and *L. turionifera* (LT).

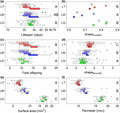

## 1 INTRODUCTION

Senescence, the progressive bodily deterioration associated with decreases in survival and reproductive output in older age classes (Kirkwood & Austad, 2000; Monaghan et al., 2008; Munne‐Bosch, 2015; Nussey et al., 2013), has been well documented in many species. For example, Jones et al. (2014) compared taxa from across the tree of life and found humans to be the greatest outliers among natural populations, exhibiting a 22‐fold increase in relative mortality (i.e., mortality measured using average life expectancy, rather than absolute time) when approaching the age of one average life expectancy. Mice (Monteforte et al., 2016; Wang et al., 2009), fish (Hsu & Chiu, 2009; Reznick et al., 2002), fruit flies (Archer et al., 2018; Grotewiel et al., 2005), honey bees (Remolina et al., 2007), nematodes (Chen et al., 2007; Lund et al., 2002), plants (Barks & Laird, 2015; Edelfeldt et al., 2019; Roach et al., 2009), and even bacteria (Ackermann et al., 2003) all experience senescence. Contrarily, certain species—typically those with an indeterminate growth form—instead demonstrate negligible or negative senescence (Baudisch et al., 2013; Vaupel et al., 2004). With so many patterns of senescence occurring throughout experimental studies and the natural world, understanding variation in these patterns has become a topic of interest across multiple disciplines, including bio‐gerontology and evolutionary biology (Hassall et al., 2017; Moorad & Promislow, 2009; Nussey et al., 2008, 2013). A key step is to develop and validate formal methods of comparing senescence patterns.

To this end, the diversity of aging patterns has spurred the development of general approaches that facilitate the comparison of aging trajectories both within and among species (Bowler & Terblanche, 2008; Jones et al., 2014; Monaghan et al., 2008). One such approach (Baudisch, 2011) aims to measure aging trajectories (i.e., population‐level patterns of survival and reproduction over time) by investigating two concepts that relate to mortality and fecundity, known as “pace” and “shape.” Pace relates to the time scale in which organisms age and is typically given as the life expectancy of a cohort of organisms of interest. Shape then quantifies how trends in survival and fecundity change over age classes by using pace as a unit of time standardization, such that time is relative to an organism's average lifespan rather than absolute (i.e., minutes, days, and years) (Wrycza et al., 2015). This approach facilitates comparisons of organisms and species that age on different time scales, which can in turn provide insight into how and why differing patterns of senescence have evolved.

Plant senescence has received less attention compared with animal senescence (Baudisch et al., 2013; Roach, 2004; Salguero‐Gomez et al., 2013). This may be due to common plant traits such as the presence of totipotent apical meristems, which may allow many plant species to escape senescence through indeterminate growth (Baudisch et al., 2013). For this reason, plant species that exhibit determinate growth may make better model organisms when studying demographic senescence. Duckweeds, the subject of the current study, are predominantly clonal plant species exhibiting determinate growth, at least at the ramet level, thus fitting this criterion.

This study implements the pace‐shape approach to compare senescence in three species of tiny, free‐floating plants in the genus *Lemna* (i.e., duckweeds): *Lemna gibba* L.*, L. minor* L., and *L. turionifera* Landolt. Individuals (i.e., ramets) are short‐lived in these species (<1 month, on average, under our lab conditions). This study complements an intraspecific comparison of duckweed, examining the patterns of aging of 27 strains of *L. turionifera* (Barks et al., 2018), which argued that intraspecific survival and fecundity trajectories were highly consistent. Our goal was to investigate whether these findings occur among congeneric species as well. Based on previous studies that found variation in survival and/or fecundity trajectories at the species and genera levels, including in plants (e.g., Dahlgren & Roach, 2017; Silvertown et al., 2001), and on previous work in our laboratory on *L. gibba*, *L. minor*, and *L. turionifera* investigated separately (Barks & Laird, 2015, 2016, 2018, Chmilar & Laird, 2019), we anticipated that there would be clearer variation in aging patterns at the species level. Notwithstanding this very general prediction, we emphasize the prospective nature of this project. In particular, at the outset, it was unclear which of the three pairs of species would have the most similar senescence patterns, with plausible arguments in favor of the more closely related pair of *L. gibba* and *L. minor* (Bog et al., 2019; Tippery & Les, 2020; Wang et al., 2010), and the more ecologically and morphologically similar pair of *L. minor* and *L. turionifera*. We report interspecific differences, but the ranking of the three species was inconsistent across the measures under examination (i.e., pace and the shapes of survival and fecundity trajectories). Nevertheless, the largest, least‐fecund, and typically longest‐lived species, *L. gibba*, tended to exhibit more rapid decreases in time‐standardized survivorship and fecundity compared with the other two species. More broadly, our work helps bring aquatic plants into the fold of comparative senescence research, which has historically had greater representation from terrestrial plants (e.g., Shefferson et al., 2017).

## 2 MATERIALS AND METHODS

### 2.1 Study species

Three duckweed species were used in this study: *L. gibba*, *L. minor*, and *L. turionifera* (family Araceae, subfamily Lemnoideae according to some sources; family Lemnaceae according to others). Duckweeds are the smallest angiosperms, found free‐floating on still or slow‐moving fresh water bodies on all continents except Antarctica (Landolt, 1986). Each of our focal species' ramets consists of a flattened “frond,” interpreted as a combination of leaf and stem tissue, with a single root protruding from the bottom surface (Lemon & Posluszny, 2000). The vegetative budding process through which duckweeds mainly reproduce occurs alternatingly at two lateral meristematic pockets. With their rapid, clonal reproduction (Ziegler et al., 2015), small size, and determinate growth at the ramet level, *Lemna* spp. are model organisms for research relating to demographic senescence, among other topics in ecology and evolution, and in other disciplines in biology (Acosta et al., 2021; Aliferis et al., 2009; Laird & Barks, 2018; Zhang et al., 2010).

*Lemna turionifera* plants used in this study were members of a colony founded from a single individual collected from a small wetland on the campus of the University of Lethbridge (strain Wat A; GenBank accession number MG000496). *Lemna minor* and *L. gibba* plants were members of lab colonies founded from plants obtained from the Canadian Phycological Culture Centre (CPCC 492, *L. minor*, GenBank accession number MG000447; CPCC 310, *L. gibba*, GenBank accession number MG000445).

### 2.2 Growth conditions and experimental design

Each frond was grown under axenic conditions in a separate 60 × 15 mm petri dish containing 10 ml of half‐strength Schenk–Hildebrandt growth medium (Sigma Aldrich S6765) supplemented with sucrose (6.7 g/L), yeast extract (0.067 g/L), and tryptone (0.34 g/L). These supplements facilitated the detection of potential microbial contaminants in the growth medium. To ensure that growth conditions were consistent throughout the experiment, each frond was transferred to fresh growth medium once per week.

Focal individuals were grown on one of four shelves with each shelf housing a random spatial arrangement of 37 individuals from each species (initial *n* = 444, the maximum that would fit while retaining a balanced design). Growth rate and lifespan in duckweed are highly temperature‐ and light‐dependent, so we chose a setup that would keep the duration of the experiment feasible. Each shelf had its own light fixture (AgroBrite FLT46), with six 122‐cm high‐output fluorescent grow bulbs (T5, 54 W, 6400 K) positioned 23.5 cm above the plants. The photoperiod was 15:9 light–dark. During the light cycle, the average photosynthetic photon flux density at plant height was approximately 410 μmol m^−2^ s^−1^, as measured with a HOBO Micro Station data logger and PAR sensor (Hoskin Scientific, Edmonton, AB). Each shelf was treated as a separate block to allow for easier division of workload (the blocks followed a staggered start) and to account for differences in environment during data analysis. The average air temperature for the light phase of each shelf was measured as: Block 1 (top shelf) = 31.2°C, Block 2 = 29.7°C, Block 3 = 27.4°C, and Block 4 (bottom shelf) = 23.6°C (since the heating environment was not recirculated, heat rose, resulting in a gradient of temperature by shelf height). The temperature for the dark phase of the cycle ranged between 20°C and 22°C across all blocks.

To keep track of focal fronds and to differentiate parental fronds from their daughters, a speck of diluted and autoclaved India ink was applied to each focal frond. To reduce the possibility of parental age effects, wherein birth order affects offspring quality (Barks & Laird, 2015, 2016), each focal frond was a descendant of a progenitor frond that had been taken from the relevant species' stock culture. Specifically, each focal individual arose from the same immediate and ancestral birth order (i.e., successive first daughters) over four generations. Each focal frond began being observed once it was “born,” defined as the day it detached from its parent, and observations ended on the day the frond was considered “dead,” defined retroactively as the day the frond's final daughter detached. Thus, “death” in this study is tantamount to the cessation of ramet production, as physiological death in duckweeds is difficult to pinpoint with any precision based on visual cues (Barks & Laird, 2015). All plants were observed daily and the number of daughters detached since the previous day was recorded (typically zero or one, and much more rarely two). Recorded daughters were discarded.

### 2.3 Sample loss

Fourteen fronds (3%) were lost during the course of the experiment due to microbial contamination or fronds growing in a clumped manner (i.e., multiple generations remaining attached and never separating, thus rendering the definition of birth inapplicable). These fronds were excluded from analysis. The final sample size was *n* = 430 (*L. gibba*: *n* = 142; *L. minor*: *n* = 147; *L. turionifera*: *n* = 141).

### 2.4 Survival and fecundity trajectories

Survival and reproduction trajectories were first analyzed separately for each of the three species (following Barks & Laird, 2015). Four parametric mortality models (exponential, Weibull, Gompertz, and logistic) were fit to the survivorship data, pooled across blocks. Log‐likelihood functions were optimized by using the *optim* function in R, and the best‐fitting model was found by calculating the Akaike Information Criterion corrected for small sample sizes (AICc). This approach minimizes the mean square error of predictions for each model, with the best‐fitting model represented by the lowest AICc value (Burnham & Anderson, 2002). The exponential model was the only model that implied no senescence, with the rate of mortality remaining unchanged with increasing age.

Generalized estimating equation (GEE) models were used to fit the proportion of individuals reproducing at a given age, again separately for each species and pooled across blocks. GEEs extend the more familiar generalized linear model approach to allow for the inclusion of longitudinal data where outcomes are correlated within subjects. This is relevant to our study, because we followed the reproduction of individuals longitudinally, and we anticipated that their daily binary reproduction scores (i.e., “reproduced” vs. “failed to reproduce”) would be correlated in time, because in duckweed, individuals may be less likely to reproduce the day after reproducing. To effect the GEE models, we used the *geeglm* function from the geepack package in R (Halekoh et al., 2006). Due to a lag in reproduction experienced by all three species (low reproductive output for the first two days), the first two days of reproduction were omitted. To model the decreased probability of an individual frond reproducing on consecutive days (i.e., to account for temporal autocorrelation), a first‐order autoregressive correlation structure was used on all three species. Fits were found for two other commonly used correlation structures (“exchangeable” and “independence”), but due to a very similar fit, these correlation structures are not included in the results.

### 2.5 Comparing pace and shape of senescence

The demographic traits compared across *Lemna* species were lifespan, shape_mortality_, total reproductive output, and shape_fecundity_. Lifespan (our pace measure) was measured as the reproductive lifespan of individual fronds, defined as the time (in days) between the day a frond detached from its parent (birth) and the day of final reproduction (death). To quantify shape_mortality_, a previously established method was used (Wrycza et al., 2015). Specifically, shape_mortality_ was measured as one minus the coefficient of variation in lifespan (1 − CV_lifespan_), where CV_lifespan_ was calculated as the standard deviation of lifespan divided by mean lifespan. Quantifying shape_mortality_ in this way allowed for straightforward categorization of aging trajectories: if mortality remained constant with age, shape_mortality_ would equal zero; if mortality increased with age, shape_mortality_ would be between zero and one, approaching one in the limiting case where all individuals died at the same age; and if mortality decreased with age, shape_mortality_ would be negative. One caveat to this approach, however, is that it cannot be applied to individual plants, as each plant only dies once (rendering the standard deviation of lifespan meaningless at the individual level). Therefore, fronds were analyzed at the cohort level to produce 12 shape_mortality_ values, one per block per species.

We note that other measures of shape_mortality_ are available, for example, those based on the Gini coefficient of lifespan or life table entropy rather than CV_lifespan_ (Wrycza et al., 2015). We chose the measure we did both because it is one of four measures deemed “suited best” according to the objective desirability criteria posited by Wrycza et al. (2015), and because it relates shape to intuitive measures of characteristic lifespan and lifespan variability (i.e., mean and standard deviation, respectively). At any rate, the measures investigated by Wrycza et al. (2015) were all highly correlated with one another when applied to empirical data.

Shape_fecundity_ was measured as the slope of the relationship between pace‐standardized age (explanatory variable) and mean‐standardized fecundity (response variable) and was based on ordinary least‐squares linear regressions applied to each plant individually, beginning at the time of first reproduction to account for the initial latency to first reproduction in duckweeds (Barks et al., 2018). Pace‐standardized age was calculated as the age of each plant divided by the mean reproductive life expectancy of that plant's species within its block, while mean‐standardized fecundity was calculated as each plant's age‐specific fecundity divided by its mean fecundity (the latter in turn calculated as the plant's total fecundity divided by its reproductive life span). Unlike shape_mortality_, shape_fecundity_ can be applied to fronds at the individual level, and a value for shape_fecundity_ was, therefore, calculated for each frond.

To test for among‐species differences in demographic traits, we used two‐way ANOVA tests including the main effects of “Species” and “Block” and their interaction (with the exception that no interaction term was modeled in the case of shape_mortality_, which had a single replicate for each Species‐Block combination). Assumptions of two‐way ANOVA were assessed with residual‐versus‐fit plots and normal quantile‐quantile plots. Log‐transformations were applied to remedy heteroscedasticity or non‐normality where appropriate. Significant species effects were accompanied by Tukey–Kramer post hoc tests to identify the nature of interspecific differences.

### 2.6 Comparing size

In addition to the demographic traits, two plant size traits were measured: frond surface area and perimeter. Photographs of each frond were taken after their final reproduction event had been recorded using a microscope‐mounted digital camera. Image analysis was conducted in MATLAB (version R2018b) using code developed by Ankutowicz and Laird (2018). We analyzed the plant size traits in the same manner as the demographic traits: with two‐way ANOVAs with log‐transformations where appropriate and with Tukey–Kramer post hoc tests.

All data were analyzed in R v. 3.6.0 (R Core Team, 2019). Data and code are archived at Dryad (Paiha & Laird, 2022).

## 3 RESULTS AND DISCUSSION

### 3.1 Survivorship and fecundity trajectories

**FIGURE 1:**
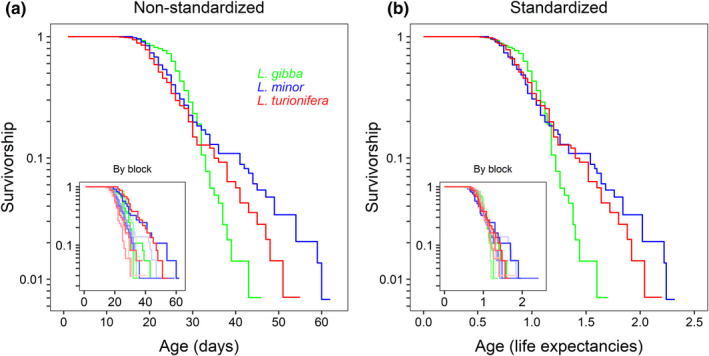
(a) Non‐standardized and (b) pace‐standardized survivorship for three species of duckweed: *Lemna gibba* = green, *L. minor* = blue, and *L. turionifera* = red (*n* = 430). Main panels show the four blocks pooled; insets show the blocks separately with lighter shades corresponding to higher‐numbered blocks

**FIGURE 2:**
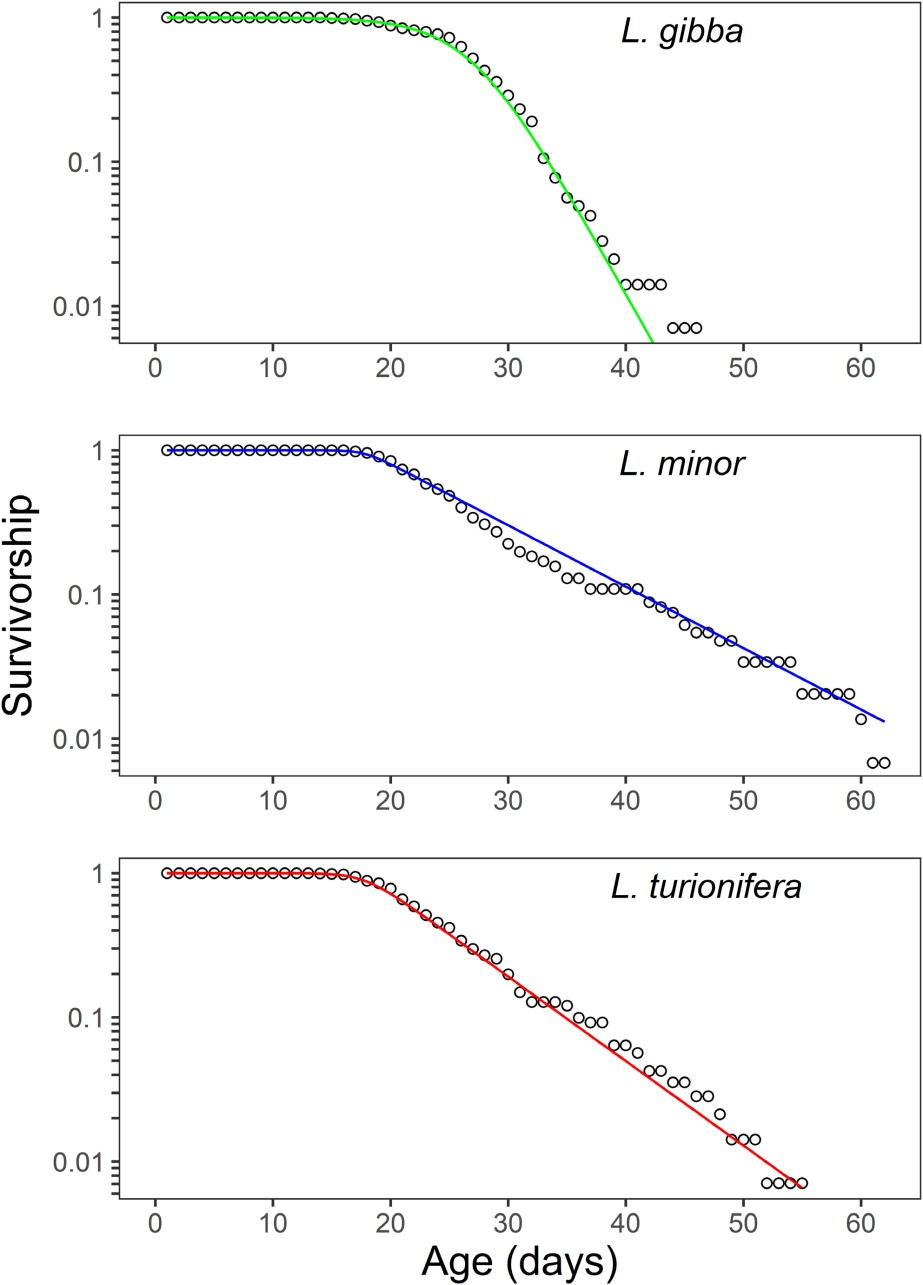
Predicted values for the best‐fitting parametric mortality model for each species (lines) and empirical values of age‐related declines in survivorship (symbols) for three species of duckweed (pooled across blocks). The best‐fitting parametric mortality model for all three species was the logistic model (see Table 1)

Age‐related declines in survivorship were observed for all three species of duckweed (Figures 1 and 2). In each case, only about 20% of fronds lived beyond age 30 days. However, the shape of each species' survivorship trajectory was distinct with *L. gibba* showing the most abrupt drop in survivorship (Figures 1a and 2), a trend that remained apparent when comparing pace‐standardized results (Figure 1b). *Lemna minor* and *L. turionifera* had more gradual decreases in survivorship and followed very similar survivorship trajectories when this measure was pace‐standardized—at least until a relative age of 1.5 times the mean life expectancy when only about 10% of fronds survived (Figure 1b). Figure 2 shows the best‐fitting parametric mortality model for each species, determined as the model with the lowest AICc value (Table 1). The logistic model was the best‐fitting parametric mortality model for all three species (Figure 2). The fact that a best‐fit model for log‐survivorship arcs downward is indicative of senescence (a lack of senescence would appear as a straight line).

**TABLE 1:** Fits of four parametric mortality models of survival (logistic, Weibull, Gompertz, and exponential) for three species of duckweed. The logistic model had the best fit for all three species (i.e., the lowest AICc)

Species	Model	Parameters	Deviance	AICc	ΔAICc
*L. gibba*	Logistic	3	882	888	0.00
	Weibull	2	894	898	9.48
	Gompertz	2	924	928	39.85
	Exponential	1	1218	1220	331.45
*L. minor*	Logistic	3	976	982	0.0
	Weibull	2	1068	1072	89.7
	Gompertz	2	1129	1133	151.3
	Exponential	1	1258	1260	278.2
*L. turionifera*	Logistic	3	918	924	0.0
	Weibull	2	980	984	60.1
	Gompertz	2	1034	1038	114.1
	Exponential	1	1188	1190	266.1

**FIGURE 3:**
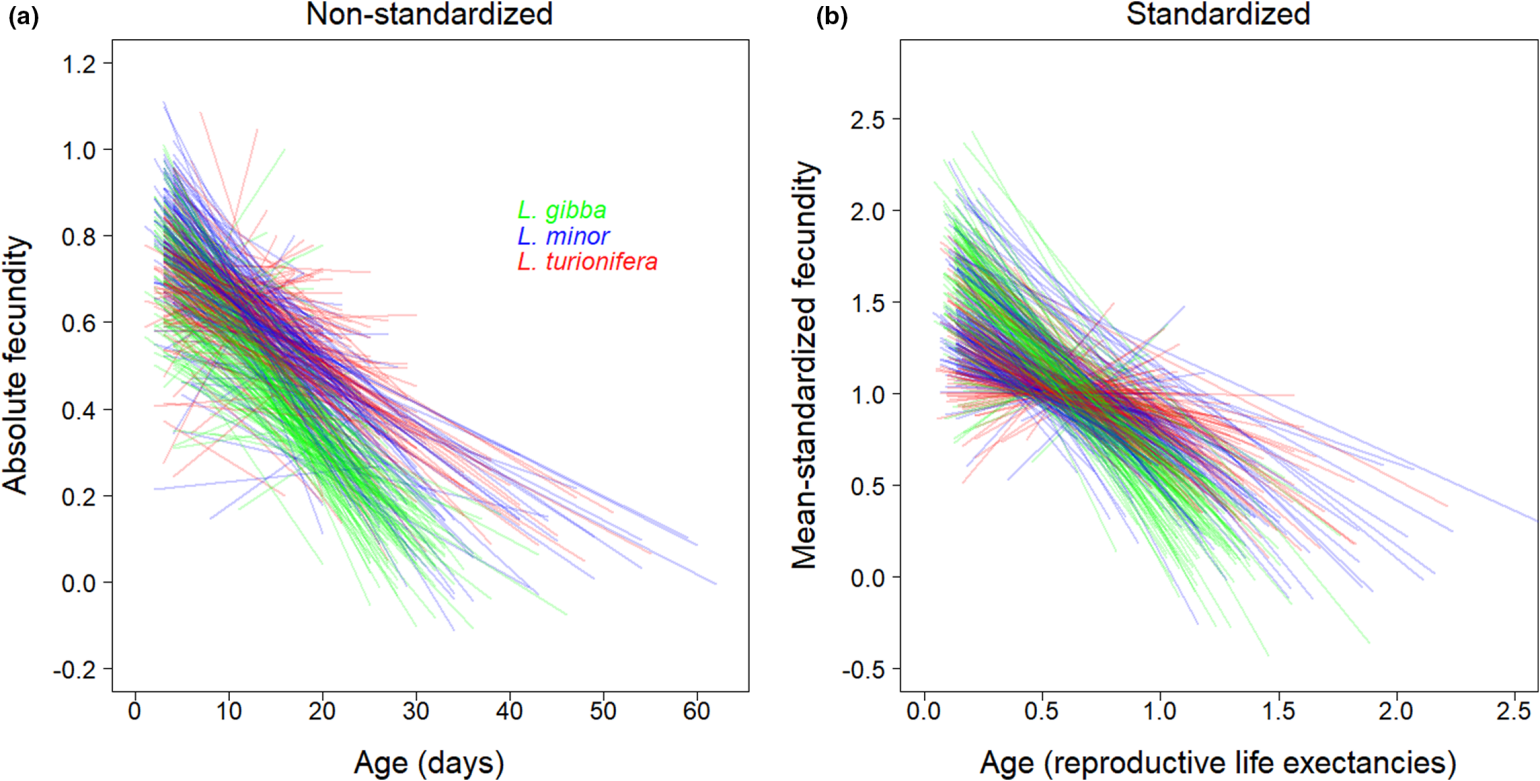
(a) Absolute fecundity versus age and (b) mean‐standardized fecundity versus pace‐standardized age: *Lemna gibba* = green, *L. minor* = blue, and *L. turionifera* = red (*n* = 430). Lines are ordinary least‐squares regressions for individuals and extend from first to last reproduction along the horizontal axes (points and indicators of block omitted to reduce clutter)

**FIGURE 4:**
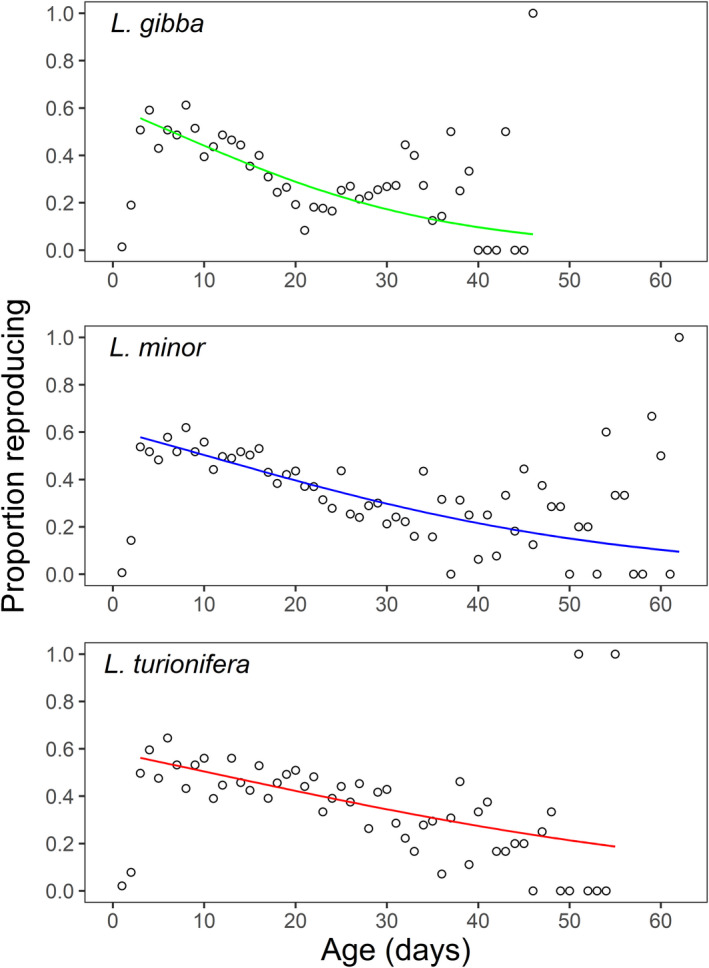
Age‐related declines in the proportion of individuals reproducing up until the maximum lifespan of each species. The best‐fit curve was fitted using a GEE model fit to each species separately. The first two days of reproduction were omitted from the GEE models as a lag in reproduction was experienced by each species. The apparent increase in variation in the proportion reproducing with age is a by‐product of the dwindling sample size caused by there being comparatively few individuals that survive to old age (see Figure 2)

In terms of fecundity, age‐related declines were also observed for all three species (Figures 3 and 4). *Lemna gibba* exhibited a more abrupt drop in absolute fecundity, while the other two species exhibited more gradual decreases. According to the fitted GEE models (Figure 4), the predicted decrease in probability of reproduction for *L. gibba* was from 0.557 at age 3 days, to 0.067 at age 46 days (maximum lifespan of longest‐lived individual). For *L. minor*, the predicted decrease was from 0.579 at age 3 days, to 0.095 at age 62 days, and for *L. turionifera* was from 0.562 at age 3 days, to 0.187 at age 55 days.

### 3.2 Pace and shape of senescence

**FIGURE 5:**
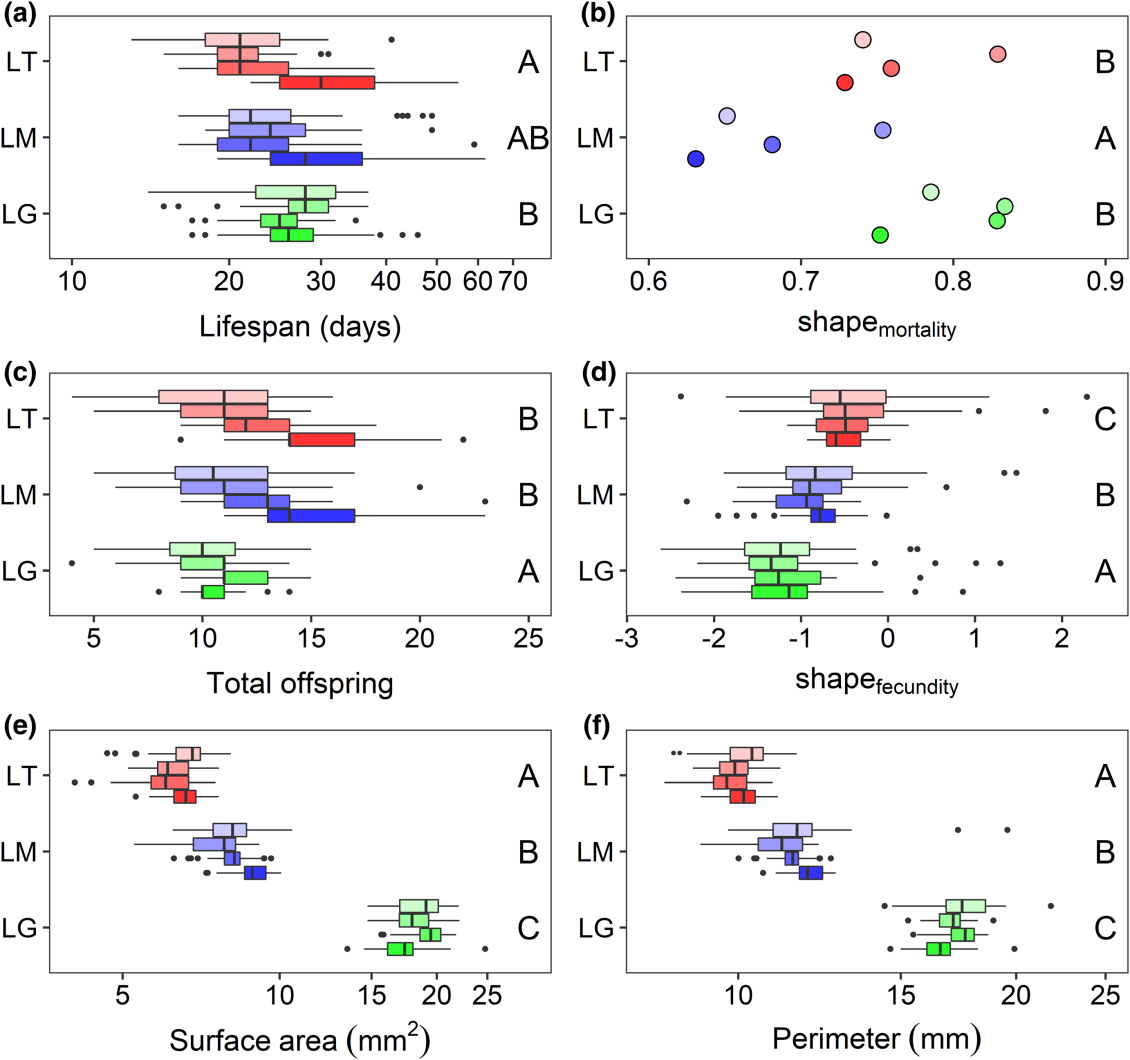
Variation in life history and size traits among three duckweed species (LG = *L. gibba* (green), LM = *L. minor* (blue), and LT = *L. turionifera* (red)). Boxes (panels (a), (c), (d), (e), (f) only) show the first, second (median), and third quartiles, while the whiskers extend to the minimum and maximum values within 1.5 times the interquartile range of the first and third quartile, respectively. The four boxes for each species (points for panel (b) that had no replication at the block level) represent the four blocks with lighter shades corresponding to higher‐numbered blocks. Letters on the right side of each panel depict the results of Tukey–Kramer post hoc tests; species with the same letter were not significantly different for the trait in question. Note the logarithmic horizontal axes in panels (a), (e), and (f), reflecting log‐transformations necessary to reduce heteroscedasticity or non‐normality in the untransformed data

Even though *L. gibba* had the shortest *maximum* lifespan, it was on average the longest‐lived species with an average lifespan of 26.8 days, followed by *L. minor* with an average lifespan of 26.6 days, and finally *L. turionifera* being the shortest‐lived species with an average lifespan of 24.8 days. There was a significant difference between the lifespans of *L. gibba* and *L. turionifera*, but not between any other pairs of species (Figure 5a and Table 2). These general trends were reflected in the warmest three blocks; however, in the coolest block, *L. minor* and *L. turionifera* had longer lifespans than *L. gibba*, reflecting the significant interaction term (Figure 5a and Table 2). Temperature dependence in duckweed lifespan has been known for more than 70 years (Wangermann & Ashby, 1951); whether, how, and why this differs among closely related duckweed species is worthy of further study.

**TABLE 2:** Two‐way ANOVAs comparing traits (“response”) among three duckweed species distributed among four blocks (note that shape_mortality_ had no replication at the block level and, therefore, has no interaction term; see *Materials and methods*). Lifespan, frond surface area, and perimeter were log‐transformed, as the untransformed data violated the assumptions of ANOVA

Response	Source	*df*	SS	MS	*F*	*p*
log(lifespan)	Species	2	0.12	0.0611	5.58	.004
	Block	3	0.58	0.1940	17.71	<.001
	Species × Block	6	0.42	0.0701	6.40	<.001
	Residual	418	4.58	0.0110		
	Total	429	5.70			
shape_mortality_	Species	2	0.0308	0.0154	52.9	<.001
	Block	3	0.0175	0.0058	20.0	.002
	Residual	6	0.00175	0.0003		
	Total	11	0.0500			
Total offspring	Species	2	353	176.3	28.3	<.001
	Block	3	715	238.3	38.2	<.001
	Species × Block	6	273	45.6	7.31	<.001
	Residual	418	2605	6.2		
	Total	429	3946			
shape_fecundity_	Species	2	35.4	17.72	52.47	<.001
	Block	3	2.8	0.94	2.77	.041
	Species × Block	6	3.1	0.52	1.53	.166
	Residual	418	141.1	0.34		
	Total	429	182.4			
log(surface area)	Species	2	16.40	8.20	3603.8	<.001
	Block	3	0.05	0.02	7.65	<.001
	Species × Block	6	0.12	0.02	8.98	<.001
	Residual	418	0.95	0.00		
	Total	429	17.52			
log(perimeter)	Species	2	4.17	2.086	2317.8	<.001
	Block	3	0.02	0.005	6.1	<.001
	Species × Block	6	0.03	0.005	5.7	<.001
	Residual	418	0.38	0.001		
	Total	429	4.60			

Reflecting their respective survivorship curves (Figure 1), *L. minor* had the lowest shape_mortality_ value, followed by *L. turionifera* and *L. gibba*; however, while *L. minor* was significantly different from the other two species, *L. turionifera* and *L. gibba* were not significantly different from each other with regard to shape_mortality_ (Figure 5b and Table 2).

*Lemna gibba* produced significantly fewer total offspring, on average, compared with the similar offspring production of *L. minor* and *L. turionifera* (Figure 5c and Table 2). Shape_fecundity_ values were significantly different among all three species (Figure 5d and Table 2), reflecting their respective fecundity trajectories, with *L. turionifera* exhibiting the largest average value for shape_fecundity_ (i.e., the shallowest decline in offspring production), followed by *L. minor*, and finally *L. gibba* exhibiting the lowest average value for shape_fecundity_ (i.e., the steepest decline in offspring production; Figure 3).

Overall, interspecific variation in pace and shape among three species of *Lemna* exceeded intraspecific variation among strains of *L. turionifera* (Barks et al., 2018). This contributes to research aimed at investigating phylogenetic patterns of senescence (Jones et al., 2014). However, it is clear that pace and shape do not strictly follow phylogenetic relatedness; for example, with regard to shape_mortality_, *L. gibba* was closer to *L. turionifera* than the more closely related *L. minor*.

While the pace‐shape approach provides a means of objectively comparing demographic trends across populations or species, a notable drawback is that it does not suggest biological mechanisms for any differences that might emerge. A key line of future research will be to investigate how variation in both the immediate environment and past selection pressures affect the pace and especially the shape of plant senescence (Barks et al., 2018; Salguero‐Gomez et al., 2013).

### 3.3 Body size

The three species showed substantial differences in size (*L. turionifera* < *L. minor* < *L. gibba*), with significant differences in both frond surface area and perimeter (Figure 5e,f and Table 2).

### 3.4 Conclusions

Cross‐species comparisons have shed some light on the many different patterns of senescence present in nature (Dudycha, 2003; Sherratt et al., 2011), and new generalized approaches have made it much easier for researchers to compare across taxonomic lines that previously were limited by differences in time scale (Baudisch et al., 2013; Jones et al., 2014). The realization that senescence is not a universal phenomenon has shifted the focus from developing theories that explain why senescence occurs toward theories explaining why there is such diversity in terms of natural patterns of senescence (Kirkwood & Austad, 2000; Wensink et al., 2017). Plants, as a specific example, have traditionally been underrepresented when it comes to research examining demographic trajectories of aging (Salguero‐Gomez et al., 2013). This study provides evidence that even closely related species can exhibit significant variation in patterns of senescence. The pace and shape approach continues to be a useful tool in the characterization of senescence trajectories in comparative studies (Archer et al., 2018; Barks et al., 2018; Baudisch, 2011).

## AUTHOR CONTRIBUTIONS

**Austin Paiha:** Conceptualization (equal); data curation (equal); formal analysis (equal); investigation (lead); methodology (equal); software (equal); validation (equal); visualization (equal); writing – original draft (lead); writing – review and editing (supporting). **Robert Laird:** Conceptualization (equal); data curation (equal); formal analysis (equal); funding acquisition (lead); investigation (supporting); methodology (equal); project administration (lead); resources (lead); software (equal); supervision (lead); validation (equal); visualization (equal); writing – original draft (supporting); writing – review and editing (lead).

## CONFLICT OF INTEREST

None declared.

## Data Availability

The data that support the findings of this study are openly available in Dryad at https://doi.org/10.5061/dryad.qv9s4mwhf.
